# Anti-MDA-5 dermatomyositis with tumid lupus-like lesions

**DOI:** 10.1016/j.jdcr.2025.02.023

**Published:** 2025-03-11

**Authors:** Kristiana Marie Jordan, Sarthak Saxena, Josephine Hai, Maxwell A. Fung, Thomas Konia, Danielle Tartar

**Affiliations:** a School of Medicine, University of California, Davis, Sacramento, California; b Department of Dermatology, University of California, Davis, Sacramento, California

**Keywords:** dermatomyositis, MDA-5, tumid lupus

## Introduction

Dermatomyositis (DM) is an idiopathic inflammatory myopathy characterized by distinctive cutaneous manifestations and a wide range of systemic manifestations.^1^ Several myositis-associated and myositis-specific autoantibodies are associated with this disease, including the antimelanoma differentiation-associated gene 5 (anti-MDA-5) antibody that is predominantly associated with clinically amyopathic dermatomyositis as well as interstitial lung disease (ILD) and even rapidly progressive ILD, which may progress to pneumomediastinum or pneumothorax.^1^^,^^2^

Previous literature demonstrates the heterogeneous nature of anti-MDA-5 DM, though certain cutaneous manifestations are associated with anti-MDA-5 DM. Palmar involvement is common, to include livedo-like findings over the digital fat pad as well as macules and papules on the interphalangeal palmar hand, oral ulcers, and alopecia.^1^ Many (60%-70%) of patients with anti-MDA-5 additionally present with classic cutaneous DM findings such as mechanic hands and Gottron papules, however, clinical presentation can differ substantially.^2^, ^3^, ^4^ There have certainly been prior reported cases of dermatomyositis presenting as cutaneous lupus,^5^, ^6^, ^7^ but to our knowledge, there are no reports of dermatomyositis presenting as tumid lupus. Tumid lupus erythematosus is a rare subset of cutaneous lupus characterized by erythematous, edematous plaques with lack of surface involvement.^8^ Overlap between the lupus erythematosus and dermatomyositis can lead to diagnostic difficulty, delayed treatment, and thus an increased risk of missing rapidly progressive ILD. In this case report, we present an uncommon case of anti-MDA-5^+^ DM presenting initially as tumid lupus erythematosus, followed closely and treated promptly with appropriate immunosuppression.

## Case

**Fig 1:**
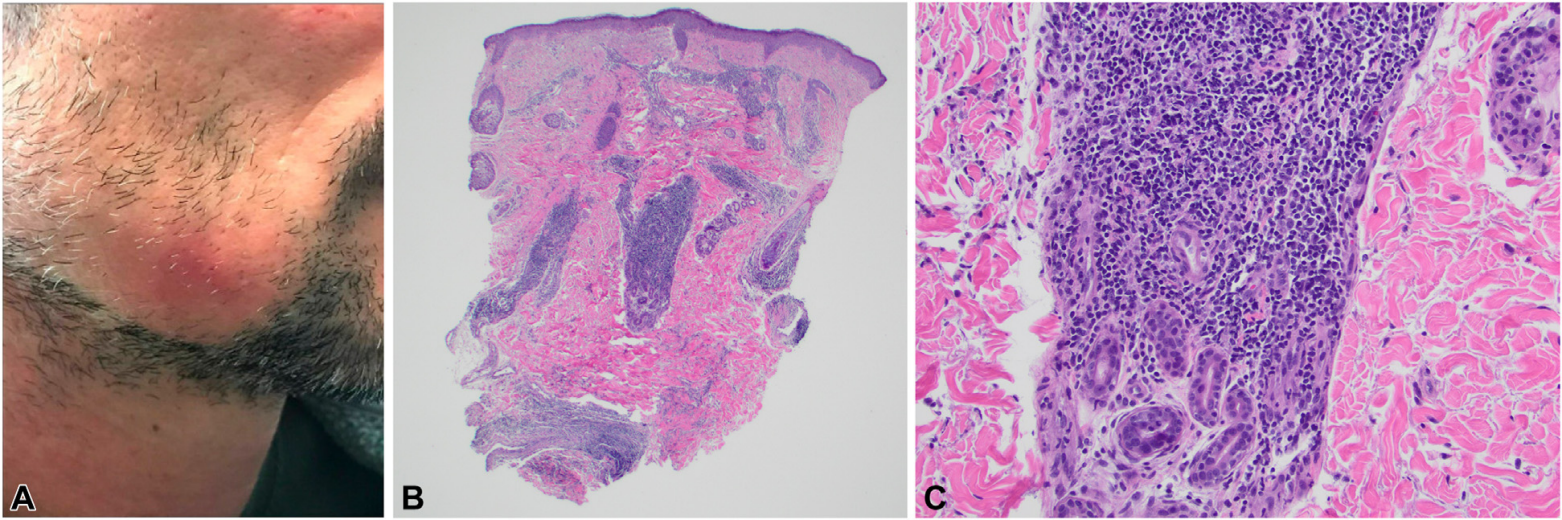
**A,** Dermatomyositis with clinical features of tumid lupus erythematosus. Right jawline with pink indurated subcutaneous plaque without epidermal changes. **B,** Dermatomyositis with histologic features of tumid lupus erythematosus. Punch biopsy of the right cheek demonstrating a moderately dense superficial and deep perivascular and periadnexal lymphocytic infiltrate. **C,** Punch biopsy of the right cheek at '40 demonstrating vacuolar alteration and rare necrotic keratinocytes along the dermoepidermal junction and superficial predominantly lymphocytic infiltrates. (**B** and **C,** Hematoxylin-eosin stain; original magnifications: **B,** ×100; **C,** ×400.)

A 32-year-old man presented with a yearlong history of redness and pain on the lower portion of the right cheek, which was noted to be photosensitive (Fig 1, *A*). Physical examination was notable for an erythematous, indurated, smooth painful plaque on the lower portion of the right cheek with overlying alopecia. Punch biopsy of the lesion revealed moderately dense superficial and deep perivascular and periadnexal predominately lymphocytic infiltrates with limited interface changes, consistent with tumid lupus (Fig 1, *B, C*). Laboratory testing was notably antinuclear antibody negative by immunofluorescence, and the patient was diagnosed with tumid lupus. He was treated with 0.1% tacrolimus ointment to the affected areas twice daily and hydroxychloroquine 200 mg twice daily. Despite strict adherence to this regimen for 4 months, he started to experience new pink, indurated, plaques without epidermal changes along the jaw, scalp, and chest similar to prior lesions and intralesional triamcinolone was injected to affected areas (5 mg/cc to the right side of chest and vertex scalp, 2.5 mg/cc to the right jaw), yielding mild improvement in pain.

**Fig 2:**
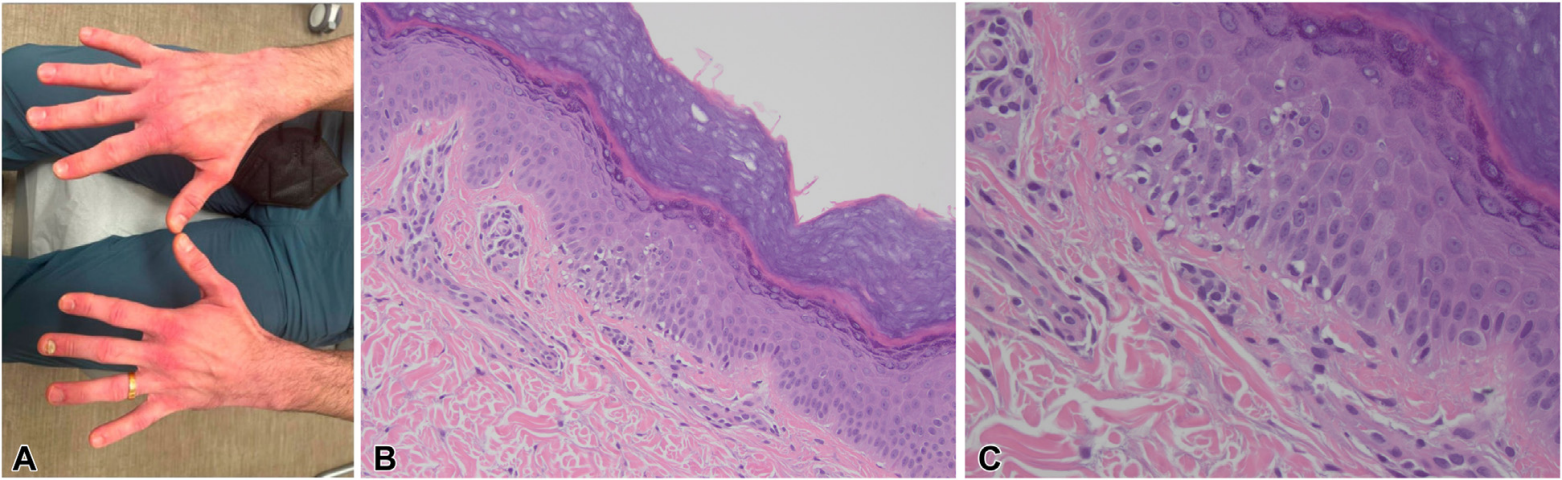
**A,** Bilateral hands with ragged cuticles and dilated capillaries on the cuticles, no Gottron papules. **B,** Interface dermatitis consistent with dermatomyositis. Punch biopsy of the dorsal aspect of the left hand demonstrating interface dermatitis. **C,** Interface dermatitis consistent with dermatomyositis. Punch biopsy of the dorsal aspect of the left hand demonstrating interface dermatitis. (**B** and **C,** Hematoxylin-eosin stain; original magnifications: **B,** ×100; **C,** ×400.)

One month later, the patient reported persistent myalgias, joint pain, alopecia, and fatigue. Hydroxychloroquine was discontinued because of suspected hypersensitivity, and the patient was asked to present to clinic for examination. Physical examination revealed rough, violaceous erythema of both dorsal aspect of the hands and lateral side of all 10 fingers, predominately over joints in addition to ragged cuticles with dilated capillaries (Fig 2, *A*). A repeat biopsy of the dorsal aspect of the left hand was performed, which revealed vacuolar alteration and rare necrotic keratinocytes along the dermoepidermal junction and superficial predominately lymphocytic infiltrates consistent with dermatomyositis (Fig 2, *B*, *C*). Additional laboratory workup revealed mildly elevated creatinine kinase (290 U/L), normal aldolase (7.4 U/L), elevated ferritin (696 U/L), and extended myositis panel was positive for MDA-5 antibodies. White blood cell count was normal (8.6 K/mm^3^) and red blood cell count was slightly decreased (4.2 M/mm^3^). Erythrocyte sedimentation rate was normal (9 mm/h). The patient was diagnosed with anti-MDA-5^+^ DM and started on a prednisone taper (starting at 1 mg/kg/d) and mycophenolate mofetil starting at 500 mg twice daily. Although pulmonary function was normal, high-resolution chest computed tomography with contrast was notable for reticular nodular opacities along the periphery of each lower lobe. The patient was promptly referred to pulmonology and rheumatology as well for comanagement.

With this immunosuppression regimen, the patient’s myalgias, fatigue, and alopecia improved. Skin lesions showed modest improvement. He was up-titrated to mycophenolate mofetil 1000 mg twice daily, monthly intravenous immunoglobulin 2 g/kg and was restarted on hydroxychloroquine 200 mg twice daily. He is also using a topical regimen consisting of alternating desonide 0.05% ointment and tacrolimus 0.1% ointment to the face daily, triamcinolone 0.1% ointment twice daily to the hands, and topical clobetasol 0.05% ointment to the trunk and extremities for flares. The patient follows with pulmonology every 6 months for management of ILD and is currently stable.

## Discussion

This case expands the clinical spectrum of dermatomyositis. In our case, the patient initially received a clinical-pathologic diagnosis of tumid lupus erythematosus, given his pink, painful, indurated subcutaneous plaques, and compatible biopsy results. Over time, our patient experienced cutaneous and systemic signs consistent with MDA-5^+^ DM, including alopecia, fatigue, myalgias, elevated ferritin, and mechanics hands and has responded well to his immune suppressive regimen. Rapidly progressive ILD occurs in 42% to 100% of patients with MDA-5^+^ DM and is characterized by irreversible, diffuse inflammation and scarring of lung tissue.^4^ If not identified and managed early in its disease course, ILD can progress to acute respiratory failure and death.^2^^,^^4^ In many cases, the progression of lung disease can outpace other symptoms, making it critical for dermatologists to hold a high index of suspicion for diagnosis, especially in patients with atypical or overlapping autoimmune symptoms.

Studies have shown that early, aggressive treatment with corticosteroids and immunosuppressive therapy can improve survival rates.^2^^,^^9^ In our case, timely administration of an immunosuppressive regimen was essential to controlling the systemic manifestations of MDA-5^+^ DM. Routine monitoring with pulmonary function tests and high-resolution computed tomography scans should be integral to follow-up care for patients with anti- MDA-5^+^ DM. Herein this case of MDA-5^+^ DM presenting as tumid lupus underscores the importance of close follow-up, early and aggressive immunosuppression, and interdisciplinary collaboration in managing this disease.

## Conflicts of interest

None disclosed.
